# Association between fear of COVID-19 and hoarding behavior during the outbreak of the COVID-19 pandemic: The mediating role of mental health status

**DOI:** 10.3389/fpsyg.2022.996486

**Published:** 2022-09-21

**Authors:** Ye Zhao, Yang Yu, Ruofan Zhao, Yiming Cai, Shuai Gao, Ye Liu, Sheng Wang, Huifeng Zhang, Haiying Chen, Youdong Li, Haishui Shi

**Affiliations:** ^1^Neuroscience Research Center, Institute of Medical and Health Science, Hebei Medical University, Shijiazhuang, China; ^2^Hebei Key Laboratory of Neurophysiology, Hebei Medicinal University, Shijiazhuang, China; ^3^Early Life Health Research Center, Hebei Medical University, Shijiazhuang, China; ^4^Nursing School, Hebei Medical University, Shijiazhuang, China; ^5^Hebei Clinical Research Center for Mental Disorders and Institute of Mental Health, Shijiazhuang, China; ^6^Department of Biochemistry and Molecular Biology, College of Basic Medicine, Hebei Medicinal University, Shijiazhuang, China

**Keywords:** COVID-19, hoarding behavior, obsessive-compulsive disorder, educational background, economic level, mental health

## Abstract

Hoarding behavior can effectively improve people's ability to resist risks, so as to reduce the negative effects of risks. However, excessive hoarding behavior will seriously reduce people's quality of life. The COVID-19 pandemic can cause excessive hoarding in a large number of people in a short period of time, and also cause a series of economic problems such as social material shortage. It is unclear how hoarding levels are linked to fear and negative emotions caused by COVID-19 among people of different educational backgrounds and social status. The purpose of this study was to explore the relationship between fear of COVID-19 and hoarding behavior in different populations in school and social contexts, as well as the mediating role of negative emotions and the moderating role of subjective/objective social status and education level in this process. An online cross-sectional survey was conducted in various provinces in China in January 2022. Demographic information, the MacArthur Scale of Subjective Social Status, the Fear of COVID-19 scale, the Depression Anxiety Stress-21, and the Saving Inventory-Revised were used to evaluate the severity of individual hoarding symptoms, the frequency of hoarding, the degree of fear, and the negative emotions (depression, anxiety, stress) caused by COVID-19. Research data showed that fear of COVID-19 was significantly correlated with hoarding behavior (*p <* 0.05). Fear of COVID-19 was significantly lower in the student sample than in the nonstudent sample (*p <* 0.05). Negative emotions played a mediating role in the relationship between fear of COVID-19 and hoarding behavior (*p <* 0.05). Educational and economic levels moderated this process, but social status did not. Compared with the student sample, educational background and income had less of a moderating effect on the depression, anxiety, and stress caused by fear of COVID-19 in the nonstudent sample. However, these factors had a more regulative effect on the clutter and excessive acquisition behavior caused by depression, anxiety, and stress, although not on difficulty discarding. These findings suggest that reduce negative emotions in the population, improve cognitive levels, and provide financial support from governments may be effective ways to reduce hoarding symptoms.

## Introduction

Hoarding is typical of many animals. Excessive hoarding is called hoarding disorder (Davidson et al., 2019). According to theory, hoarding disorder arises from unusual beliefs regarding and strong emotional attachment to items as well as executive functioning deficits, behavioral avoidance, and early developmental factors (Frost and Hartl, 1996; Mataix-Cols et al., 2010). Recently, the fifth edition of the Diagnostic and Statistical Manual of Mental Disorders officially classified hoarding disorder as a disease, which is associated with trichotillomania and body dysmorphic disorder. Hoarding disorder refers to "unrestrained accumulation behavior," which is characterized by individuals’ tendency to constantly acquire and accumulate new items in their living quarters and to experience difficulty discarding useless hoarded items or even excessive demand or other behaviors. This behavior makes it impossible for such people to live a normal life (Battle, 2013). Worryingly, an assessment of hoarding behaviors among Asian adults found a lifetime prevalence of 2% and a general prevalence of 22.6% (Subramaniam et al., 2014). Hoarding disorder has seriously reduced the quality of life of this segment of the population and significantly affected the average emotional level of patients.

Some cities in China continued to report higher numbers of COVID-19 cases in January. In response, China has adopted a unique and efficient dynamic clearing strategy for the domestic pandemic. China's stringent zero-COVID strategy will face its toughest test when millions of people travel around the country for Chinese New Year and the Winter Olympics begin in Beijing. The government's dynamic clearing of COVID-19 has inevitably led to blockades of some areas (Xiong et al., 2020; Mallapaty, 2022). Similar to the situation of the SARS epidemic in China in 2003 (Maunder et al., 2003; Chan et al., 2006; Cheung et al., 2008). Our study was conducted in this epidemic environment, the intermittent shortages of living materials and individual social isolation caused by COVID-19 may have negative impacts on individual mental health that cannot be ignored. COVID-19 significantly impacts people’s risk perceptions, stress levels, and fear. In terms of risk perceptions, one study used the 8-item COVID-19 Risk Perception Scale to assess individual risk associated with COVID-19 (Yıldırım and Güler, 2020). The results showed that people’s levels of risk perception are significantly positively correlated with the severity of COVID-19 and self-efficacy. This assessment was conducted by using a scale related to exposure to stressors (Tambling et al., 2021), which showed that people's stress increased significantly during the COVID-19 pandemic. In terms of fear, Chung-Ying Lin et al. developed the Fear of COVID-19 Scale (FCV-19S) to assess the population's fear of COVID-19 during the pandemic, which has been associated with satisfactory psychological measurement results in 10 countries (Lin et al., 2021). In addition, experimental data have shown that during the COVID-19 pandemic, fear of the pandemic has increased significantly. Recently, studies have used the Obsessive Compulsive Inventory-Revised, the Cognitive and Affective Mindfulness Scale-Revised, and the Depression Anxiety Stress Scales-21 (DASS-21) to evaluated the general population (Marazziti et al., 2021) and found that the prevalence of obsessive-compulsive hoarding disorder increased by approximately 4% following the onset of the pandemic.

Recent studies have reported a significant increase in the hoarding of daily necessities such as food, drinking water, and toilet paper during the pandemic (Benatti et al., 2020; Sim et al., 2020). The frequency of this phenomenon and the increasing number of people with hoarding disorder have drawn attention from academic circles. Since the end of 2019, studies have mainly focused on the relationship between the dissemination of information by news media, social distancing, personality traits, stress levels, negative emotion levels and hoarding behavior during the COVID-19 pandemic. A study of 770 adolescents in the United States (Oosterhoff and Palmer, 2020) showed that the more frequently they were exposed to COVID-19-related media and disinfection practices, the higher their degree of hoarding behavior. In addition, 530 Japanese adults (Yoshino et al., 2021) and the German population (Fink et al., 2021) were evaluated in terms of hoarding behavior and personality traits. Multivariate regression analysis showed that individuals with high levels of agreeableness, neuroticism, openness, and cupidity tended to hoard more items during the pandemic. Individuals experience higher levels of depression, anxiety and distress in response to stressful events related to COVID-19 and may exhibit greater degrees of hoarding disorder (Fontenelle and Miguel, 2020; Ouellette et al., 2021). Importantly, some research data have shown that obsessive-compulsive hoarding places a tremendous burden on an individual's economic level (Tolin et al., 2010). Groups of different social classes and economic levels have different susceptibility to stress and different tolerance to risk (Cheng and Chan, 2008). However, the question of whether there is a correlation between the hoarding levels of people from different educational backgrounds and the fear and anxiety caused by COVID-19 remains under investigation, and the influence of social status and economic level on this process remains unclear. Therefore, this paper used a scale to evaluate the relationship between negative emotions and hoarding behavior during the COVID-19 pandemic as well as the mediating and moderating roles played by educational background and economic level in this process, with the aim of providing a reference concerning the impact of COVID-19 on individual psychological emotions and hoarding behavior.

## Materials and methods

### Participants

Over the course of nearly one month (10 January 2022 to 10 February 2022), offline survey activities and personal freedoms were restricted due to the requirements of the relevant governments (for example, large-scale events were banned, public facilities were closed, and people were urged to reduce social contact). In addition, policies related to pandemic prevention and control prevented offline survey activities from being carried out normally. Therefore, we distributed a questionnaire aimed at evaluating hoarding behavior to the subjects via online and social media channels. An a priori sample size calculation for *t*-test design with an anticipated medium effect size of 0.5, power level of 0.95, and *p* < 0.05 for two groups and seven measurement times (Faul et al., 2007), suggested a minimum sample size of 210 participants (G*Power 3.1.9.6 software; Faul et al., 2007). And an a priori sample size calculation for correlation analysis design with an anticipated medium effect size of 0.3, power level of 0.9, and *p* < 0.05 for two groups and seven measurement times, suggested a minimum sample size of 109 participants. (Bäuerle et al., 2020a,b; Teufel et al., 2020; Weismüller et al., 2020). In the analysis, we included only individuals aged 18–65, excluding younger and older respondents as well as respondents whose answer times were too short or too long. All participants provided written informed consent prior to participating in the online survey. The survey was completely anonymous and was approved by the relevant ethics committee.

### Assessment

We use an online software named “Questionnaire Star” to design the questionnaire content and publish the questionnaire online. This study used qualitative methods to collect data, and the entire evaluation process took about 180–600 s. The estimated time is based on the fastest time for 15 undergraduate volunteers to fill out the questionnaire effectively. In addition, considering that a long answer time may affect the questionnaire quality, we invited mental health experts from Hebei Medical University to conduct an evaluation. Finally, we set the upper limit of the answer time as 600 s. During this assessment, participants were able to save their responses and double-check their responses upon submission. The assessment asked respondents to report demographic information such as their age, gender, living area and place of birth, educational background, subject type, whether or not they were an only child, whether or not they were a student, their objective economic condition and social status level. The MacArthur Scale of Subjective Social Status (Goodman et al., 2007), the Saving Inventory-Revised (SI-R) (Frost et al., 2004, 2011; Tolin et al., 2010; Kellman-McFarlane et al., 2019), the Depression Anxiety Stress Scale-21 (DASS-21) (Henry and Crawford, 2005; Bäuerle et al., 2020a,b; Hetkamp et al., 2020; Musche et al., 2020; Weismüller et al., 2020; Schweda et al., 2021; Skoda et al., 2021a,b) the Fear of COVID-19 Scale (FCV-19S) (Bäuerle et al., 2020a,b; Hetkamp et al., 2020; Musche et al., 2020; Weismüller et al., 2020; Schweda et al., 2021; Skoda et al., 2021a,b), and the Hoarding Frequency Scale were also included. The collected data were analyzed by using Pearson correlation analysis, a confidence test, an independent sample *t-*test, a regression analysis, and mediating and moderating effect analyses. To ensure the accuracy of the analysis, all scales in this study refer to authoritative journal literature, and mature scales with high reliability and efficiency are selected. All scales were developed by foreign scholars and the original scale language was English. The Fear of COVID-19 scale, the Saving Inventory-Revised etc. were translated and backtranslated according to the procedure proposed by Brislin (Davidson et al., 2019). Our team invited 3 bilingual doctors to translate and reverse translate the scale, and invited 2 experts in the field of psychology to investigate the scale and adjust it according to the situation.

### Demographic information

In terms of demographic information, the questionnaire assessed the participants’ age, gender, place of living and place of birth, educational background (the educational background of the subjects was evaluated on a 3-point scale; 1 = junior college, 2 = undergraduate, 3 = master’s degree or above), major, whether or not the participants was an only child, whether or not the participant was a student, participants’ objective economic condition and social status level, namely, the occupation of the participant’s father/mother (subjects' parents’ occupation was evaluated on a 5-point scale; 1 = temporary worker, unemployed, farmer or unskilled worker, etc., 2 = manual worker, such as construction worker, technician, self-employed and related personnel, 3 = general management personnel, technicians, commercial service personnel such as a driver, salesperson, etc., 4 = middle management personnel, personnel of state organs, party and government organizations, personnel of public institutions, scientific and technical personnel such as a teacher, doctor, etc. 5 = senior management personnel, party and government organs of leading cadres, private owners such as a civil servant, manager, etc.), level of education (father/mother divided in accordance with scores concerning standard assessment subjects with 6 levels of education for parents; 1 = elementary school or primary school, 2 = junior high school degree, 3 = high school or technical secondary school degree, 4 = college degree, 5 = bachelor's degree, 6 = graduate degree or above), and annual family income (RMB was used as the economic standard to evaluate the economic conditions of the subjects on a 10-point scale, 1 = no income, 2 = 2,000 RMB or less, 3 = 2,000–5,000 RMB, 4 = 5,000–10,000 RMB, 5 = 10,000–30,000 RMB, 6 = 30,000–50,000 RMB, 7 = 50,000–100,000 RMB, 8 = 100,000–150,000 RMB, 9 = 150,000–200,000 RMB, 10 = 200,000 RMB and above).

#### MacArthur scale of subjective social status

This scale is a self-report tool. The scale features a single dimension and includes only one item. It is used as a subjective evaluation of the social status, social status, and economic level of both students and nonstudents as well as the degrees of visibility and respect, reputation and prestige associated with those individuals (Goodman et al., 2007). The scale is scored on a 10-point scale. The higher the corresponding score selected by the subject is, the higher the subject’s level social status according to his or her subjective evaluation.

#### Saving inventory-revised

This inventory is a self-report tool used to assess the severity of hoarding disorder in subjects (Frost et al., 2004). The scale includes 21 items, which are divided into three dimensions: clutter, difficulty discarding, and excessive acquisition. The original SI-R has been proven to exhibit good psychometric characteristics in terms of internal consistency, confidence, convergence and divergence (Tolin et al., 2010; Frost et al., 2011). The total score of the scale ranges from 0 to 92, with a 5-point scale ranging from 0 (never) to 4 (extremely) for each item. The higher the subject's assessment score is, the more likely he or she is to engage in the behavior in question. Recently, Kellman-McFarlane et al. proposed 39 points as the optimal threshold to distinguish hoarders from nonhoarders by reference to the receiver operating characteristic curve (Kellman-McFarlane et al., 2019).

#### Depression anxiety stress scale-21

This scale is a self-report tool used to assess participants' levels of negative emotions such as depression, anxiety and stress. The scale consists of 21 items divided into 3 dimensions in accordance with Clark and Watson's tripartite model (Henry and Crawford, 2005), namely, depression (sadness, anhedonia, lack of initiative, low self-esteem, etc.), anxiety (panic, fear, etc.) and stress (irritability, impatience, tension, etc.). Experience has proven that DASS-21 exhibits good psychometric characteristics in various environments (Henry and Crawford, 2005). The total score of the scale is 0–63 points (0–21 points for each dimension), and the score for each item is 0–3 points (always matched). The higher the evaluation score of the subject is, the higher the corresponding levels of depression, anxiety and stress.

#### Fear of COVID-19 scale

This scale is a self-report tool used to assess subjects’ level of fear during the COVID-19 pandemic. Following a revision, the scale consists of 7 items and a single dimension. The original FCV-19S has been shown to exhibit good psychometric characteristics in various situations (Bäuerle et al., 2020a,b; Hetkamp et al., 2020; Musche et al., 2020; Weismüller et al., 2020; Schweda et al., 2021; Skoda et al., 2021a,b). Each item is scored on a 5-point scale ranging from 1 (strongly disagree) to 5 (strongly agree). The higher the subject's assessment score is, the stronger that subject’s level of fear of the novel coronavirus.

#### Hoarding frequency scale (from store)

This scale is a self-report tool used to assess hoarding frequency via a single item that asked respondents how often they hoarded supplies from a grocery store or department store over the past 7 days. The Hoarding Frequency Scale features a single dimension and only one item, and it has been shown to exhibit good psychometric characteristics in various environments (Oosterhoff and Palmer, 2020). The total score of the scale ranged from 1 to 5, and each item is scored from 1 (never) to 5 (very frequently). The higher the evaluation score of the subject is, the more frequently that subject exhibits hoarding behavior.

#### Statistical analysis

After entering the data into the computer, we used SPSS 21.0 software for data analysis. The steps are as follows: First, the deviation analysis of common methods; Secondly, descriptive statistics and Pearson correlation analysis were carried out for the main variables. Third, the *t*-test was used to determine the differences in relevant indicators between student samples and nonstudent samples, and between hoarders and nonhoarders. Fourthly, the process plug-in developed by Hayes is used to select models 7, 8, 14, 15, 58, 59 to test the mediation model and moderation model regulation model respectively. Descriptive statistics are reported as mean +/− standard deviation. Independent sample *t-*test is to divide the samples into student samples and nonstudent samples, as well as into hoarder samples and nonhoarder samples for analysis. We planned a mediation and moderation analysis with bootstrapping techniques using the PROCESS macro for SPSS (version 3.0; Hayes, 2015). Overall, we performed six models, using consistently the total FCV-19S score as an independent variable. SI-R (Clutter, Difficulty Discarding and Excessive Acquisition) and Hoarding Frequency Scale scores served as dependent variables in separately calculated models. DASS-21 (Anxiety, Depression, and Stress) total score and subscale score were used as mediating variables. We performed 10,000 bootstrap samples to generate a 95% bias-corrected confidence interval of the indirect effect a*b. In our mediation analysis, the a path represented the path from FCV-19S to DASS-21, and the b path represented the path from DASS-21 to SI-R and Hoarding Frequency Scale. The output from our model also included path c, the path from FCV-19S to SI-R and Hoarding Frequency Scale. Additionally, we conducted a moderation model with total FCV-19S score serving as an independent variable, while SI-R and Hoarding Frequency Scale total scores were used again as dependent variables in separately calculated models. In this model, educational background, economic level and social status are moderating variables.

## Results

### Description of the sample

Statistics are described in terms of percentages, the mean and standard deviation for a normal distribution curve, and median and range (Min–Max) for a nonnormal distribution curve. All the subjects were from China (*n* = 643, 100%), and the average age of participants was 25.28 (8.03) years old. In terms of education, at least 545 participants (84.76%) had a bachelor's degree or higher level of education, with the average score being 1.94 (0.49). In terms of age, the mean age of subjects who were assessed as hoarders (*n* = 187) was 25.37 (7.21) years. Among the samples, the mean total score of the evaluated SI-R was 33.14 (11.89), the mean total score on DASS-21 was 12.15 (11.39), and the mean total score on FCV-19S was 17.56 (6.15). The average score on the Hoarding Frequency Scale was 2.39 (0.89). In addition, participants’ average economic level was 6.45 (1.95). The specific clinical data of the subjects are summarized in Table 1.

**Table 1:** Summary of participants’ clinical features.

	Means (SD)	Cut-off scores	% of individuals at the clinical level
Saving inventory-revised	33.14 (11.89)	≥41 (Frost et al., 2004; Tolin et al., 2011)	29.08%
Clutter	12.15 (5.64)	≥17 (Frost et al., 2004; Tolin et al., 2011)	22.40%
Difficulty discarding	10.59 (3.80)	≥14 (Frost et al., 2004; Tolin et al., 2011)	22.86%
Excessive acquisition	10.40 (3.94)	≥9 (Frost et al., 2004; Tolin et al., 2011)	67.81%
Depression anxiety stress scale-21	12.15 (11.39)	–	–
Depression	3.69 (4.09)	>9 (Frost et al., 2011)	9.18%
Anxiety	3.70 (3.83)	>7 (Frost et al., 2011)	14.62%
Stress	4.76 (4.12)	>14 (Frost et al., 2011)	2.02%
Fear of COVID-19 scale	17.55 (6.15)	–	–
Hoarding frequency scale	2.39 (0.89)	–	–
Educational background	1.94 (0.49)	–	–
Economic level	6.45 (1.95)	–	–

### Common method bias test of variables

The results of the principal component factor analysis showed that 12 factors in the total samples featured a characteristic grounding greater than 1 and that the variation explained by the first factor was only 23.51%, i.e., less than 40% of the critical standard, there is no evidence of a common method bias.

### Correlation analysis among scales

Pearson correlation analysis of each scale and the demographic information showed that educational background was significantly correlated with scores on the DASS-21 total scale and subscale, FCV-19S and Hoarding Frequency Scale. The McArthur subjective social status scale was only correlated significantly with economic level and educational background. The DASS-21 total scale and subscale were significantly correlated with the SI-R total scale and subscale. In addition, the FCV-19S and Hoarding Frequency Scale were significantly correlated with the other scales. The correlation analysis among the specific scales is shown in Table 2.

**Table 2:** Summary of the analysis of correlations among the scales (*r*).

	1	2	3	4	5	6	7	8	9	10	11	12
Educational background (1)												
Economic level (2)	<0.01^**^											
Clutter (3)	0.29	0.06										
Difficulty discarding (4)	0.44	0.05	<0.01^**^									
Excessive acquisition (5)	0.05	0.07	<0.01^**^	<0.01^**^								
Saving inventory-revised (6)	0.17	0.03^*^	<0.01^**^	<0.01^**^	<0.01^**^							
Depression (7)	<0.01^**^	0.01^*^	<0.01^**^	<0.01^**^	<0.01^**^	<0.01^**^						
Anxiety (8)	<0.01^**^	<0.01^**^	<0.01^**^	<0.01^**^	<0.01^**^	<0.01^**^	<0.01^**^					
Stress (9)	0.01^*^	<0.01^**^	<0.01^**^	<0.01^**^	<0.01^**^	<0.01^**^	<0.01^**^	<0.01^**^				
Depression anxiety stress scale-21 (10)	<0.01^**^	<0.01^**^	<0.01^**^	<0.01^**^	<0.01^**^	<0.01^**^	<0.01^**^	<0.01^**^	<0.01^**^			
Fear of COVID-19 scale (11)	<0.01^**^	0.5	<0.01^**^	<0.01^**^	<0.01^**^	<0.01^**^	<0.01^**^	<0.01^**^	<0.01^**^	<0.01^**^		
Hoarding frequency scale (12)	0.02^*^	0.09	<0.01^**^	<0.01^**^	<0.01^**^	<0.01^**^	<0.01^**^	<0.01^**^	<0.01^**^	<0.01^**^	<0.01^**^	
MacArthur scale of subjective social status (13)	0.04^*^	<0.01^**^	0.83	0.77	0.35	0.61	0.13	0.95	0.12	0.26	0.28	0.12

* *p* < 0.05, calculated using 2-tailed bivariate correlations;

** *p* < 0.01, calculated using 2-tailed bivariate correlations.

#### Comparison of scores on various scales between students and nonstudents

An independent samples *t-*test analysis was conducted on the scale data for both students and nonstudents. Statistical data showed that MacArthur Scale of Subjective Social Status scores, total FCV-19S scores and economic level of school students were significantly lower than those of nonstudents, educational background scores were significantly higher for students than nonstudents, and Fear of COVID-19 Scale scores were significantly lower for students than nonstudents. No significant differences were found with respect to the other demographic data or scale and subscale scores. The comparison data of the scores on all the scales are summarized in Table 3.

**Table 3:** Summary of data for each scale score comparison (*x* ± *s*).

Parameter	Students (*N* = 438)	Nonstudents (*N* = 205)	*t*	*p*
MacArthur scale of subjective social status	4.30 ± 1.56	5.36 ± 1.97	–6.753	<0.01^**^
Clutter	12.16 ± 5.50	12.12 ± 5.92	0.099	0.92
Difficulty discarding	10.73 ± 3.65	10.30 ± 4.10	1.326	0.19
Excessive acquisition	10.45 ± 3.74	10.30 ± 4.32	0.421	0.67
Saving inventory-revised	33.34 ± 11.27	32.72 ± 13.14	0.584	0.56
Depression	3.78 ± 4.12	3.50 ± 4.05	0.811	0.42
Anxiety	3.86 ± 3.91	3.35 ± 3.64	1.580	0.11
Stress	4.78 ± 4.21	4.72 ± 3.94	0.183	0.86
Depression anxiety stress scale-21	12.42 ± 11.59	11.57 ± 10.97	0.888	0.38
Fear of COVID-19 scale	17.15 ± 5.76	18.41 ± 6.84	–2.28	0.02^*^
Hoarding frequency scale	2.35 ± 0.91	2.47 ± 0.84	–1.52	0.13
Educational background	1.99 ± 0.43	1.84 ± 0.60	3.11	<0.01^**^
Economic level	6.29 ± 1.95	6.77 ± 1.92	–2.869	<0.01^**^

* *p* < 0.05, calculated using 2-tailed bivariate correlations;

** *p* < 0.01, calculated using 2-tailed bivariate correlations.

#### Comparison of scale scores for hoarders and nonhoarders

**Figure 1:**
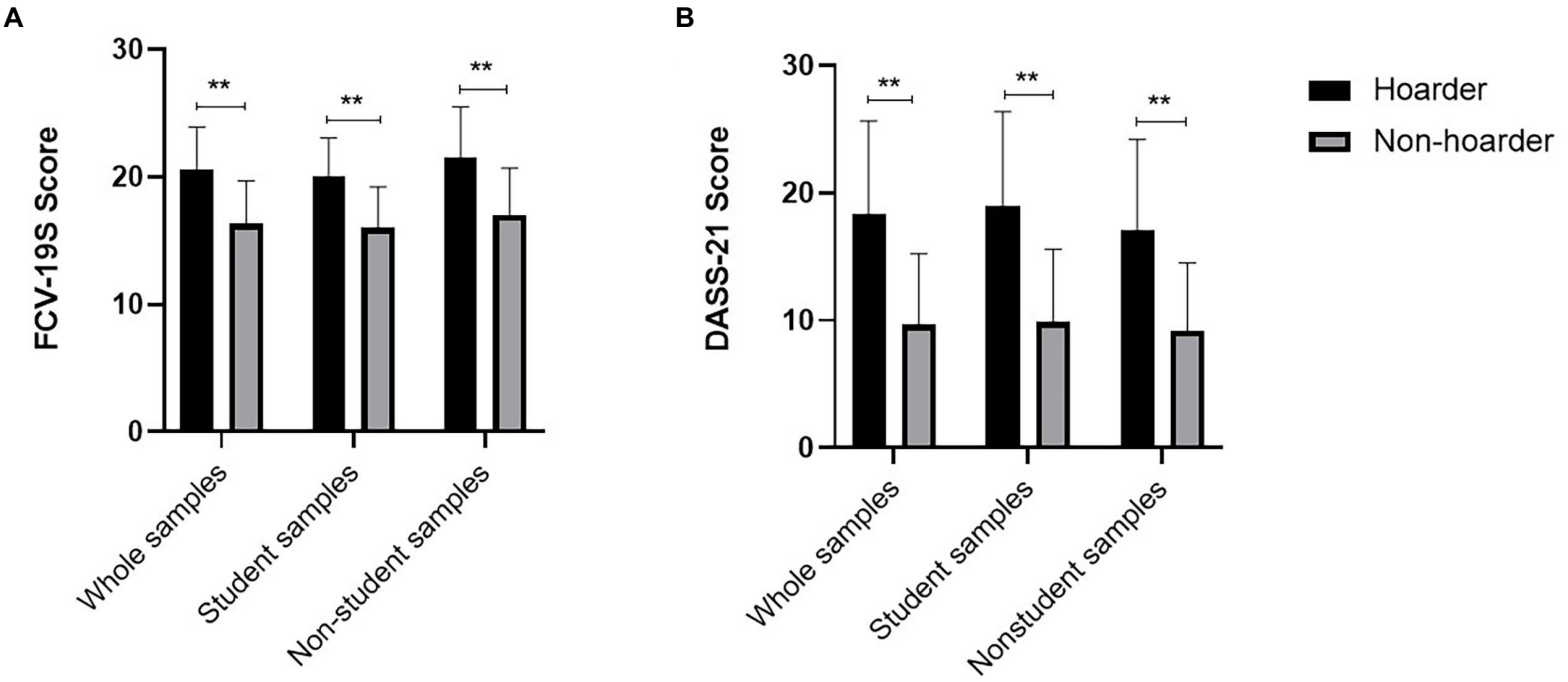
**(A)** Histogram model of the comparison of FCV-19S scores between hoarders and nonhoarders. **(B)** Histogram model of the comparison of DASS-21 scores between hoarders and nonhoarders. The difference between the two scores was statistically significant (^**^p < 0.01).

According to the scoring criteria suggested by Frost et al., we divided all samples into hoarders and nonhoarders by reference to the SI-R scale with a critical value of 41. Independent sample *t-*test analysis was conducted on the data of each scale for both hoarders and nonhoarders. The statistical results showed that the scores of DASS-21, its subscale and FCV-19S were significantly higher for hoarders than for nonhoarders (*p* < 0.01) and that the difference in these scores was statistically significant. No significant differences were found for the demographic data or scale and subscale scores. The comparison of scores between DASS-21 and FCV-19S is shown in Figure 1.

### Hierarchical linear regression analysis of SI-R, DASS-21 and FCV-19S

As shown in Tables 4, 5, SI-R is strongly correlated with DASS-21 and FCV-19S scores. With respect to fear of the COVID-19 pandemic in the context of the magnitude and frequency of hoarding behavior during this process, we further investigate whether DASS-21 scores play a mediating role in this process for all participants. Statistical analysis of the available data for all the samples demonstrates that DASS-21 scores regarding clutter and excessive acquisition constitute a significant portion of the mediating effect. The specific multiple linear regression analysis is summarized in Tables 4, 5.

**Table 4:** Results of mediation analysis (clutter).

	Clutter					DASS-21					Clutter				
	B	SE	t	*p*	**β**	B	SE	t	*p*	**β**	B	SE	t	*p*	**β**
Constant	7.166^**^	0.64	11.194	<0.01	–	0.185	1.266	0.146	0.884	–	7.139^**^	0.613	11.641	<0.01	–
FCV-19S	0.284^**^	0.034	8.249	<0.01	0.31	0.682^**^	0.068	10.017	<0.01	0.368	0.184^**^	0.035	5.195	<0.01	0.201
DASS-21											0.146^**^	0.019	7.643	<0.01	0.296
*R* ^2^	0.096					0.135					0.172				
Adjust *R*^2^	0.095					0.134					0.169				
*F*	F(1,641) = 68.042					*F*(1,641) = 100.336					*F*(2,640) = 66.274				
Value	*p* < 0.01					*p* < 0.01					*p* < 0.01				

* *p* < 0.05, calculated using 2-tailed bivariate correlations;

** *p <* 0.01, calculated using 2-tailed bivariate correlations.

**Table 5:** Results of mediation analysis (excessive acquisition).

	Excessive acquisition					DASS-21					Excessive acquisition				
	B	SE	t	*p*	**β**	B	SE	t	*p*	**β**	B	SE	t	*p*	**β**
Constant	6.626^**^	0.443	14.962	<0.01	–	0.185	1.266	0.146	0.884	–	6.604^**^	0.415	15.9	<0.01	–
FCV-19S	0.215^**^	0.024	9.024	<0.01	0.336	0.682^**^	0.068	10.017	<0.01	0.368	0.132^**^	0.024	5.479	<0.01	0.206
DASS-21											0.122^**^	0.013	9.428	<0.01	0.354
*R* ^2^	0.113					0.135					0.221				
Adjust *R*^2^	0.111					0.134					0.218				
*F*	*F* (1,641) = 81.432					*F* (1,641) = 100.336					*F* (2,640) = 90.738				
Value	*p* < 0.01					*p* < 0.01					*p* < 0.01				

* *p* < 0.05, calculated using 2-tailed bivariate correlations;

** *p* < 0.01, calculated using 2-tailed bivariate correlations.

### An analysis of the moderating effect of level of education and income in on increasing hoarding behavior induced by fear of COVID-19 as mediated by DASS-21

**Figure 2:**
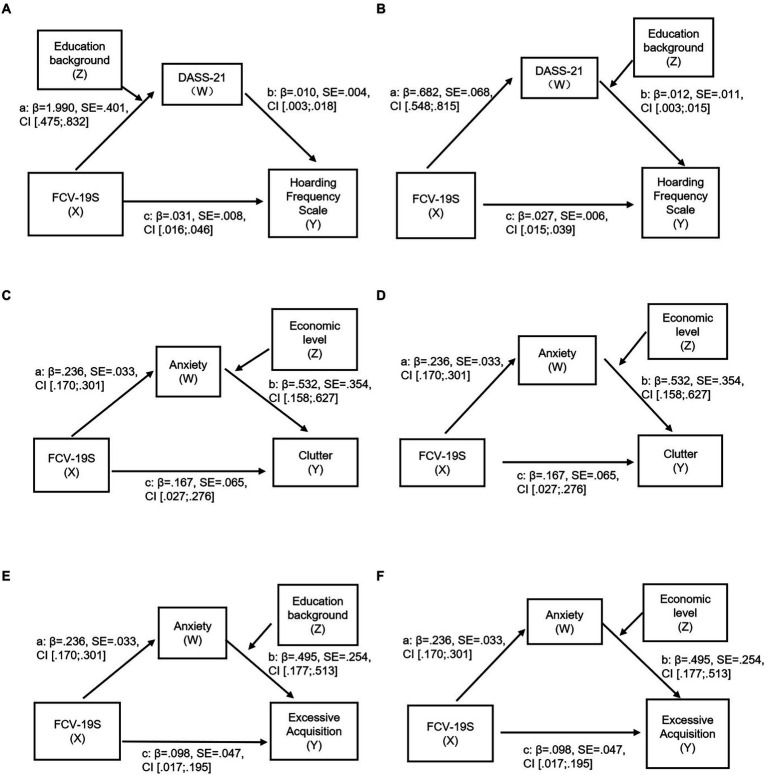
**(A)** DASS-21 mediating and moderating model of the correlation between FCV-19S scores and Hoarding Frequency Scale scores. c is the direct effect of FCV-19S scores and Hoarding Frequency Scale scores; a and b are the fully normalized indirect effects of DASS-21 (student sample). **(B)** DASS-21 mediating and moderating model of the correlation between FCV-19S scores and Hoarding Frequency Scale scores. c is the direct effect of FCV-19S scores and Hoarding Frequency Scale scores; a and b are the fully normalized indirect effects of DASS-21 scores (overall sample). **(C,D)** Anxiety mediating and moderating model of the correlation between FCV-19S scores and clutter. c is the direct effect of FCV-19S scores and clutter; a and b are the fully normalized indirect effects of anxiety (nonstudent sample). **(E,F)** Anxiety mediating and moderating model of the correlation between FCV-19S scores and excessive acquisition. c is the direct effect of FCV-19S scores and excessive acquisition; a and b are the fully normalized indirect effects of anxiety (nonstudent sample).

Based on the results of the correlation analysis and multiple linear regression analysis of this study (as shown above), we selected the overall sample, student sample and nonstudent sample to explore the mediating and moderating effects of other scales and variables on the SI-R, DASS-21, FCV-19S and Hoarding Frequency Scale in further detail. According to the analysis of the mediating and moderating effects of level of education and income, the statistical results show that these factors have a significant moderating effect on multiple models of the Hoarding Frequency Scale as a dependent variable among both the student and overall samples, but not among the nonstudent sample (Figures 2A,B). Compared with the student sample, the educational background and income of the nonstudent sample had less of a moderating effect on the depression, anxiety and stress caused by fear of COVID-19. However, this variable has a more regulative effect on the clutter and excessive acquisition behavior caused by depression, anxiety and stress, but not on difficulty discarding (Supplementary Tables S1–S6). In this context, fear of COVID-19 may have a particular effect on the nonstudent sample via anxiety (Figures 2C–F). The analysis of the important mediating and moderating effects of level of education income among the entire sample, the student sample and the nonstudent sample is shown in Figure 2.

## Discussion

In this study, an online questionnaire was used to explore the impact of COVID-19 on the mood and hoarding behavior of individuals from different educational backgrounds. In addition, the method of manual intervention was used to review the quality of individuals’ answers to facilitate the selection of answers and improve efficiency. Ultimately, a total of 643 valid samples were obtained (after eliminating questionnaires that took less than 180 s to complete). To the best of our knowledge, this was the first study to use the DASS-21 mediation model and educational, economic, and subjective social status to investigate whether the association between FCV-19S and SI-R can be partially explained by the psychological effects of the COVID-19 pandemic. Two main assessment findings are reported. First, the level of education of the population sample, college student sample and professional sample, using the Hoarding Behavior Scale or SI-R as the dependent variable, has a significant moderating effect on the various models, but the sample’s level of social status does not. Second, DASS-21 significantly mediates the effect of FCV-19S on SI-R and Hoarding Behavior Scale scores. Our research can facilitate the development of targeted knowledge publicity and psychotherapy programs for individuals from different social strata, with different incomes and from different educational backgrounds. This approach can reduce the mass panic caused by COVID-19 and the shortage of supplies caused by excessive hoarding, thereby reducing the delivery pressure faced by the material transportation system during the pandemic.

The impact of fear of the COVID-19 pandemic on physical and psychological stress responses has received a great deal of attention. Studies have shown that COVID-19 has increased people's anxiety, depression, insomnia, hoarding and other behaviors and has even aggravated these characteristics into mental diseases, thereby seriously harming people's physical and mental health (Fontenelle and Miguel, 2020; Fontenelle et al., 2021; Kim et al., 2021; Franklin et al., 2022).

Our questionnaire design was the first to quantify the correlations among people's fear of COVID-19, negative emotions (depression, anxiety and stress), educational background, income, social status and hoarding behavior. Our analysis of the entire sample showed that people's fear of COVID-19 was positively correlated with negative emotions (depression, anxiety and stress), which is similar to the conclusions of previous studies. Our study was the first to discover that academic degree is negatively correlated with fear of COVID-19, negative emotions (depression, anxiety and stress), and hoarding frequency. This result may be related to the higher cognitive level of highly educated individuals, such as certain knowledge related to disease prevention and control and health literacy (Li and Liu, 2020). This knowledge enables highly educated individuals to react to the negative news caused by the novel coronavirus in a more rational manner and to exhibit greater ability to engage in information screening (Kim et al., 2021), which can effectively reduce the possibility of individuals blindly hoarding daily necessities. Simultaneously, highly educated individuals may have a high degree of understanding of the transmission vectors of COVID-19 and can better comply with personal protective measures (Bazaid et al., 2020). In addition, highly educated individuals can continue to maintain a high frequency of exercise during the closures caused by the pandemic (de Boer et al., 2021), and such a high frequency of exercise may make it easier for these individuals to release and relieve negative emotions, thereby allowing them to exhibit fewer symptoms such as depression and anxiety (Wolf et al., 2021). There are negative correlations among family income, hoarding behavior and negative emotions (depression, anxiety and stress). That is, high family income may offer individuals more material security and a stronger ability to withstand the risks caused by the blockade, such that high-income individuals do not need to worry about the decline in quality of life caused by economic problems (Bierman et al., 2021). Furthermore, such a level of income mitigates the influence of life pressure caused by insufficient income that can lead to individual depression, anxiety and other symptoms (Ettman et al., 2020). Interestingly, subjective social status is not associated with hoarding behavior or negative emotions, and higher income does not reduce COVID-19 fear to the same degree as higher education. Therefore, it can be speculated that increasing residents' income can reduce individual hoarding behavior, but reducing people's psychological fear of COVID-19 nevertheless requires the improvement of the overall cognitive level nationwide.

Subsequently, we further split the overall sample into student samples and nonstudent samples. Following a comparative analysis of the scale data between the two samples, it can be concluded that the economic level and subjective social status of the student sample are significantly higher than those of the nonstudent sample during the COVID-19 period. Our analysis suggests that this finding may be due to the different environments faced by students and nonstudents. People who are not on campus are frequently employed and live off campus and can move freely within a certain area, while the activity areas of on-campus students are usually limited to the campus (Mallapaty, 2022). In addition, these groups face different risks from COVID-19, since people who are not in school are frequently quarantined, unable to work and lose income after contracting or coming into close contact with COVID-19. In-school students who become infected or experience close contact with COVID-19 are also treated in isolation. Unlike those who are not in school, in-school students do not face pressure regarding income, and they can continue to study for their specialized courses normally via the internet. In this study, the subjective social rank and fear of COVID-19 of nonstudents were significantly higher than those reported by current students, which is consistent with the expected results. In the sample of students, educational background, family income and subjective social status were not correlated with hoarding behavior. Among these factors, level of education only had an impact on fear of COVID-19 and negative emotions, and among nonstudents, level of education was only negatively correlated with fear of COVID-19 fear, while family income was more closely correlated with hoarding behavior and negative emotions (depression, anxiety and pressure). A degree may entail a higher level of knowledge and ability, indicating that for students in school, attainment of a degree can reduce their fear of COVID-19.

Studies have shown that fear of COVID-19 can cause anxiety, depression and other mental diseases (Fitzpatrick et al., 2020; Nicomedes and Avila, 2020; Stein, 2020; Millroth and Frey, 2021; Ypsilanti et al., 2021). Reducing people's fear of COVID-19 is important not only to improve their ability to cope but also to reduce rates of depression and anxiety.

Our study used analyses of mediating and moderating effects to explore the relationships among COVID-19 fear, severity of psychological distress (anxiety, depression, and stress), and hoarding severity. Mediation analysis refers to a series of methods aimed at extracting causal mechanism information concerning the influence of predictors on results (Mokhayeri and Mansournia, 2019). We also took into account individuals’ subjective social status and educational backgrounds and found that the severity of psychological distress has a partial mediating effect on the hoarding behavior caused by fear of COVID-19 in the whole sample. To our knowledge, no prior studies have explored the mediating patterns operative among individual fear of COVID-19, severity of psychological distress, and hoarding severity. This research can facilitate a better understanding of the relationship between negative emotions in communities and abnormal hoarding behavior in the context of the COVID-19 pandemic, which can be reduced by decreasing people's fear of COVID-19 and their levels of depression and anxiety. Interestingly, after we analyzed the mediating and moderating effects in the context of the subscale on the nonstudent sample, we found that depression, anxiety and stress played a mediating role in the excessive acquisition behavior and accumulation behavior caused by fear of COVID-19. In addition, fear of COVID-19 in particular may have a specific effect on nonstudent samples via anxiety. Among these mediating effects, educational background and income play a moderating role in the process by which depression, anxiety and stress promote an increase in excessive seeking and accumulation behavior, respectively (Supplementary Tables S1–S6). In this context, depression, anxiety, and stress do not, for the most part, mediate the process of COVID-19 fear-induced difficulty discarding. This result could be due to people’s focus on buying large quantities of necessities due to the quarantines and lockdowns instituted in response to the-COVID-19 pandemic. Such anxious and panic-related buying behavior is the main driving factor of the accumulation of items and excessive demand (Teufel et al., 2020), but it is unrelated to difficulty discarding. In other words, during the pandemic, people tend to focus on buying behaviors to alleviate the anxiety and stress resulting from the COVID-19 pandemic (Tse et al., 2022), while less attention is given to the discarding of everyday items. The moderating effect of educational background and income may be due to the fact that individuals with superior educational backgrounds generally have higher levels of awareness of measures related to pandemic prevention and control (Bazaid et al., 2020; Li and Liu, 2020; Li et al., 2021), which can prevent the blind buying and hoarding behaviors caused by anxiety, pressure and other emotional factors. High income can greatly reduce the impact of the pandemic on daily life, allowing individuals to maintain a high level of quality of life without anxiety that can promote an increase in hoarding behavior (Xiong et al., 2020; Huang et al., 2022). It is worth noting that compared with the student sample, educational background and income in the nonstudent sample have lesser moderating effects on the depression, anxiety and stress resulting from fear of COVID-19 and greater moderating effects on the excessive acquisition behavior and clutter behavior caused by depression, anxiety and stress.

Although the findings are interesting, some limitations still exist in the present study. During the survey period, the COVID-19 epidemic situation and related prevention and control policies are changing rapidly, and the epidemic trend and policy change are easy to affect the public's emotional level, thus reducing the accuracy of the survey results. Therefore, we choose to shorten the survey time to 3 days to minimize the instability of the psychological and emotional level and hoarding level of the survey population. In addition, the sample size will be further reduced after the sample is divided into student samples and nonstudent samples. However, according to the data analyzed, the sample size of N = 643 is also sufficient to reflect and express the correlation between COVID-19 fear and hoarding behavior among the current domestic population. Our findings therefore suggest a new strategy: governments could raise awareness of COVID-19 by encouraging media campaigns, or by targeting subsidies to those disproportionately affected by the pandemic. These measures have a positive moderating effect on improving hoarding behavior indirectly caused by fear of contracting COVID-19. However, the depression, anxiety and stress caused by fear of COVID-19 must also be managed in other ways, such as counseling by a professional counselor or taking medication for mental illness.

## Data availability statement

The raw data supporting the conclusions of this article will be made available by the authors, without undue reservation.

## Ethics statement

The studies involving human participants were reviewed and approved by Medical Ethics Committee of Hebei Medical University. Written informed consent for participation was not required for this study in accordance with the national legislation and the institutional requirements.

## Author contributions

HS: conceptualization, methodology, supervision, resources, and review and editing. YZ: conceptualization, methodology, data curation, supervision, and review and editing. YY: conceptualization, methodology, data curation, writing-original draft, formal analysis, and writing-review and editing. RZ: conceptualization, methodology, data curation, and formal analysis. YC, SG, YLiu, SW, HZ, HC, and YLi: conceptualization, methodology, and data curation. All authors contributed to the article and approved the submitted version.

## Funding

This study was partly financed by the National Natural Science Foundation of China (82171536 and 81771462), the Innovative Research Group Project of Hebei Province Natural Science Foundation (H2021206203), and the Hebei Medical University College Students Innovative Experiment Project (USIP2022074).

## Conflict of interest

The authors declare that the research was conducted in the absence of any commercial or financial relationships that could be construed as a potential conflict of interest.

## Publisher’s note

All claims expressed in this article are solely those of the authors and do not necessarily represent those of their affiliated organizations, or those of the publisher, the editors and the reviewers. Any product that may be evaluated in this article, or claim that may be made by its manufacturer, is not guaranteed or endorsed by the publisher.

## Supplementary material

The Supplementary material for this article can be found online at: https://www.frontiersin.org/articles/10.3389/fpsyg.2022.996486/full#supplementary-material
