# Supplementary material for: Association between fear of COVID-19 and hoarding behavior during the outbreak of the COVID-19 pandemic: The mediating role of mental health status
Source: Front Psychol. 2022 Sep 21;13:996486. doi: 10.3389/fpsyg.2022.996486 (PMC9534310; doi:10.3389/fpsyg.2022.996486)
Supplement: Supplementary file 1 [file Table_1.DOC]

Supplementary information

# Supplementary Tables

**Table.S1**

Results of moderated mediation effect analysis (FCV-19S =>Stress =>Clutter, Education background)

|  | Clutter | | | | Stress | | | |
| --- | --- | --- | --- | --- | --- | --- | --- | --- |
|  | β | SE | t | p | β | SE | t | p |
| Constant | 4.448 | 2.315 | 1.921 | 0.056 | -0.681 | 0.683 | -0.996 | 0.32 |
| FCV-19S | 0.157 | 0.069 | 2.273 | 0.024^*^ | 0.293 | 0.035 | 8.426 | ＜0.01^**^ |
| Education background | 1.79 | 1.037 | 1.726 | 0.086 |  |  |  |  |
| Stress | 0.749 | 0.321 | 2.333 | 0.021^*^ |  |  |  |  |
| Stress*Education background | -0.237 | 0.165 | -1.44 | 0.152 |  |  |  |  |
| sample capacity | 205 | | | | 205 | | | |
| R ² | 0.128 | | | | 0.259 | | | |
| Adjust R ² | 0.106 | | | | 0.252 | | | |
| F  Value | F (4,200) =7.335  p＜0.01 | | | | F (1,203) =70.993  p＜0.01 | | | |

| Intervening variable | Mean | Value | Effect | BootSE | BootLLCI | BootULCI |
| --- | --- | --- | --- | --- | --- | --- |
| Stress | Low Mean（-1SD） | 1.246 | 0.133 | 0.046 | 0.039 | 0.225 |
|  | Mean | 1.844 | 0.091 | 0.038 | 0.013 | 0.164 |
|  | High Mean  (+1SD) | 2.442 | 0.05 | 0.052 | -0.063 | 0.144 |

Footnote: ^*^ P < 0.05, calculated using 2-tailed bivariate correlations. ^**^P < 0.01, calculated using 2-tailed bivariate correlations.BootLLCI refers to the lower limit of 95% interval of Bootstrap sampling, and BootULCI refers to the upper limit of 95% interval of Bootstrap sampling.

**Table.S2**

Results of moderated mediation effect analysis (FCV-19S =>Stress =>Clutter, Economic level)

|  | Clutter | | | | Stress | | | |
| --- | --- | --- | --- | --- | --- | --- | --- | --- |
|  | β | SE | t | p | β | SE | t | p |
| Constant | 8.879 | 2.78 | 3.193 | 0.002^**^ | -0.681 | 0.683 | -0.996 | 0.32 |
| FCV-19S | 0.144 | 0.068 | 2.121 | 0.035^*^ | 0.293 | 0.035 | 8.426 | ＜0.01^**^ |
| Economic level | -0.137 | 0.341 | -0.401 | 0.689 |  |  |  |  |
| Stress | 0.358 | 0.415 | 0.863 | 0.389 |  |  |  |  |
| Stress*Economic level | -0.006 | 0.061 | -0.093 | 0.926 |  |  |  |  |
| sample capacity | 205 | | | | 205 | | | |
| R ² | 0.117 | | | | 0.259 | | | |
| Adjust R ² | 0.095 | | | | 0.252 | | | |
| F  Value | F (4,200) =6.657  p＜0.01 | | | | F (1,203) =70.993  p＜0.01 | | | |

| Intervening variable | Mean | Value | Effect | BootSE | BootLLCI | BootULCI |
| --- | --- | --- | --- | --- | --- | --- |
| Stress | Low Mean（-1SD） | 4.842 | 0.097 | 0.052 | 0.003 | 0.204 |
|  | Mean | 6.766 | 0.094 | 0.037 | 0.019 | 0.166 |
|  | High Mean（+1SD） | 8.689 | 0.091 | 0.054 | -0.017 | 0.193 |

Footnote: ^*^ P < 0.05, calculated using 2-tailed bivariate correlations. ^**^P < 0.01, calculated using 2-tailed bivariate correlations.BootLLCI refers to the lower limit of 95% interval of Bootstrap sampling, and BootULCI refers to the upper limit of 95% interval of Bootstrap sampling.

**Table.S3**

Results of moderated mediation effect analysis (FCV-19S =>Stress =>Excessive Acquisition, Education background)

|  | Excessive Acquisition | | | | Stress | | | |
| --- | --- | --- | --- | --- | --- | --- | --- | --- |
|  | β | SE | t | p | β | SE | t | p |
| Constant | 6.797 | 1.638 | 4.149 | ＜0.01^**^ | -0.681 | 0.683 | -0.996 | 0.32 |
| FCV-19S | 0.064 | 0.049 | 1.312 | 0.191 | 0.293 | 0.035 | 8.426 | ＜0.01^**^ |
| Education background | 0.341 | 0.734 | 0.465 | 0.643 |  |  |  |  |
| Stress | 0.709 | 0.227 | 3.123 | ＜0.01^**^ |  |  |  |  |
| Stress*Education background | -0.191 | 0.117 | -1.642 | 0.102 |  |  |  |  |
| sample capacity | 205 | | | | 205 | | | |
| R ² | 0.181 | | | | 0.259 | | | |
| Adjust R ² | 0.16 | | | | 0.252 | | | |
| F  Value | F (4,200) =11.018  p＜0.01 | | | | F (1,203) =70.993  p＜0.01 | | | |

| Intervening variable | Mean | Value | Effect | BootSE | BootLLCI | BootULCI |
| --- | --- | --- | --- | --- | --- | --- |
| Stress | Low Mean（-1SD） | 1.246 | 0.138 | 0.033 | 0.077 | 0.208 |
|  | Mean | 1.844 | 0.104 | 0.03 | 0.049 | 0.164 |
|  | High Mean（+1SD） | 2.442 | 0.071 | 0.038 | -0.007 | 0.144 |

Footnote: ^*^ P < 0.05, calculated using 2-tailed bivariate correlations. ^**^P < 0.01, calculated using 2-tailed bivariate correlations.BootLLCI refers to the lower limit of 95% interval of Bootstrap sampling, and BootULCI refers to the upper limit of 95% interval of Bootstrap sampling.

**Table.S4**

Results of moderated mediation effect analysis (FCV-19S =>Stress =>Excessive Acquisition, Economic level)

|  | Excessive Acquisition | | | | Stress | | | |
| --- | --- | --- | --- | --- | --- | --- | --- | --- |
|  | β | SE | t | p | β | SE | t | p |
| Constant | 7.711 | 1.956 | 3.941 | ＜0.01^**^ | -0.681 | 0.683 | -0.996 | 0.32 |
| FCV-19S | 0.085 | 0.048 | 1.775 | 0.077 | 0.293 | 0.035 | 8.426 | ＜0.01^**^ |
| Economic level | -0.082 | 0.24 | -0.344 | 0.731 |  |  |  |  |
| Stress | 0.596 | 0.292 | 2.041 | 0.043^*^ |  |  |  |  |
| Stress*Economic level | -0.04 | 0.043 | -0.923 | 0.357 |  |  |  |  |
| sample capacity | 205 | | | | 205 | | | |
| R ² | 0.18 | | | | 0.259 | | | |
| Adjust R ² | 0.159 | | | | 0.252 | | | |
| F  Value | F (4,200) =10.971  p＜0.01 | | | | F (1,203)=70.993  p＜0.01 | | | |

| Intervening variable | Mean | Value | Effect | BootSE | BootLLCI | BootULCI |
| --- | --- | --- | --- | --- | --- | --- |
| Stress | Low Mean（-1SD） | 4.842 | 0.118 | 0.039 | 0.048 | 0.2 |
|  | Mean | 6.766 | 0.096 | 0.028 | 0.043 | 0.154 |
|  | High Mean（+1SD） | 8.689 | 0.073 | 0.038 | -0.001 | 0.148 |

Footnote: ^*^ P < 0.05, calculated using 2-tailed bivariate correlations. ^**^P < 0.01, calculated using 2-tailed bivariate correlations.BootLLCI refers to the lower limit of 95% interval of Bootstrap sampling, and BootULCI refers to the upper limit of 95% interval of Bootstrap sampling.

**Table.S5**

Results of moderated mediation effect analysis (FCV-19S =>Depression =>Excessive Acquisition, Economic level)

|  | Clutter | | | | Depression | | | |
| --- | --- | --- | --- | --- | --- | --- | --- | --- |
|  | β | SE | t | p | β | SE | t | p |
| Constant | 8.363 | 2.283 | 3.663 | ＜0.01^**^ | -0.671 | 0.753 | -0.891 | 0.374 |
| FCV-19S | 0.149 | 0.062 | 2.415 | 0.017^*^ | 0.226 | 0.038 | 5.903 | ＜0.01^**^ |
| Economic level | -0.055 | 0.276 | -0.198 | 0.843 |  |  |  |  |
| Depression | 0.598 | 0.405 | 1.479 | 0.141 |  |  |  |  |
| Depression*Economic level | -0.031 | 0.06 | -0.524 | 0.601 |  |  |  |  |
| sample capacity | 205 | | | | 205 | | | |
| R ² | 0.146 | | | | 0.147 | | | |
| Adjust R ² | 0.125 | | | | 0.138 | | | |
| F  Value | F (4,200) =8.565  p＜0.01 | | | | F (1,203) =34.846  p＜0.01 | | | |

| Intervening variable | Mean | Value | Effect | BootSE | BootLLCI | BootULCI |
| --- | --- | --- | --- | --- | --- | --- |
| Depression | Low Mean（-1SD） | 4.842 | 0.101 | 0.044 | 0.022 | 0.193 |
|  | Mean | 6.766 | 0.088 | 0.031 | 0.032 | 0.152 |
|  | High Mean（+1SD） | 8.689 | 0.074 | 0.043 | -0.008 | 0.163 |

Footnote: ^*^ P < 0.05, calculated using 2-tailed bivariate correlations. ^**^P < 0.01, calculated using 2-tailed bivariate correlations.BootLLCI refers to the lower limit of 95% interval of Bootstrap sampling, and BootULCI refers to the upper limit of 95% interval of Bootstrap sampling.

**Table.S6**

Results of moderated mediation effect analysis (FCV-19S =>DASS-21 =>Excessive Acquisition, Education background)

|  | Clutter | | | | DASS-21 | | | |
| --- | --- | --- | --- | --- | --- | --- | --- | --- |
|  | β | SE | t | p | β | SE | t | p |
| Constant | 4.959 | 2.225 | 2.229 | 0.027^*^ | -2.343 | 1.95 | -1.202 | 0.231 |
| FCV-19S | 0.145 | 0.066 | 2.188 | 0.030^*^ | 0.756 | 0.099 | 7.607 | ＜0.01^**^ |
| Education background | 1.558 | 0.989 | 1.576 | 0.117 |  |  |  |  |
| DASS-21 | 0.265 | 0.114 | 2.316 | 0.022^*^ |  |  |  |  |
| DASS-21*Education background | -0.069 | 0.06 | -1.145 | 0.253 |  |  |  |  |
| sample capacity | 205 | | | | 205 | | | |
| R ² | 0.146 | | | | 0.222 | | | |
| Adjust R ² | 0.124 | | | | 0.214 | | | |
| F  Value | F (4,200) =8.517  p＜0.01 | | | | F (1,203) =57.861  p＜0.01 | | | |

| Intervening variable | Mean | Value | Effect | BootSE | BootLLCI | BootULCI |
| --- | --- | --- | --- | --- | --- | --- |
| DASS-21 | Low Mean（-1SD） | 1.246 | 0.135 | 0.044 | 0.051 | 0.226 |
|  | Mean | 1.844 | 0.104 | 0.036 | 0.034 | 0.175 |
|  | High Mean（+1SD） | 2.442 | 0.073 | 0.051 | -0.036 | 0.168 |

Footnote: ^*^ P < 0.05, calculated using 2-tailed bivariate correlations. ^**^P < 0.01, calculated using 2-tailed bivariate correlations.BootLLCI refers to the lower limit of 95% interval of Bootstrap sampling, and BootULCI refers to the upper limit of 95% interval of Bootstrap sampling.
